# 1929. Evaluating Disparities in Recurrent *Clostridioides difficile* Infection (CDI) and Fecal Microbiota Transplant (FMT) Treatment using Geospatial and Social Vulnerability Analytic Tools

**DOI:** 10.1093/ofid/ofad500.089

**Published:** 2023-11-27

**Authors:** Keighly Little, Nirja Mehta, Dana Goodenough, Robin Dhonau, Nitin Gupta, Michael H Woodworth, Scott Fridkin

**Affiliations:** Georgia Emerging Infections Program, Decatur, GA; Emory University School of Medicine, Atlanta, GA; Atlanta Veterans Affairs Medical Center, Decatur, GA, Atlanta, Georgia; Emory University, Atlanta, GA; Georgia Emerging Infections Program, Decatur, GA; Emory University School of Medicine, Atlanta, GA; Atlanta Veterans Affairs Medical Center, Decatur, GA, Atlanta, Georgia; Georgia Emerging Infections Program, Decatur, GA; Emory University School of Medicine, Atlanta, GA; Atlanta Veterans Affairs Medical Center, Decatur, GA, Atlanta, Georgia; Atlanta Gastroenterology Associates, Atlanta, Georgia; Emory University, Atlanta, GA; Georgia Emerging Infections Program, Decatur, GA; Emory University School of Medicine, Atlanta, GA, Atlanta, Georgia

## Abstract

**Background:**

Recurrent CDI (rCDI) can be deadly and live biotherapies such as fecal microbiota transplantation (FMT) are now recommended at 2^nd^ recurrence. Although incidence of Clostridioides difficile infection (CDI) historically is higher in white compared to black patients, the role of race or social determinates of health on rCDI is unclear. This study explores social determinants of risk for rCDI and treatment with FMT.

**Methods:**

Georgia’s Emerging Infections Program (EIP) (supported by CDC) conducts CDI surveillance in 8 metropolitan Atlanta counties. Incident CDI (+toxin or molecular assay) in resident of catchment area (without previous + within 14 days) between 2016 and 2019 were categorized as single episode or rCDI ( >1 episode within 365 days) and geocoded (ArcGIS)-linked to CDC’s 2020 Social Vulnerability Index (SVI) data. Numerical values of each SVI variable (Table 1) were assigned to each patient. SVI theme and composite SVI were assessed as predictors for each rCDI and FMT receipt with most vulnerable defined categorically (top quartile) in log binomial logistic regression. Spatial analysis produced spot map of FMT, and choropleth maps of the CDI and rCDI rates per 100,000 persons.

**Figure:**
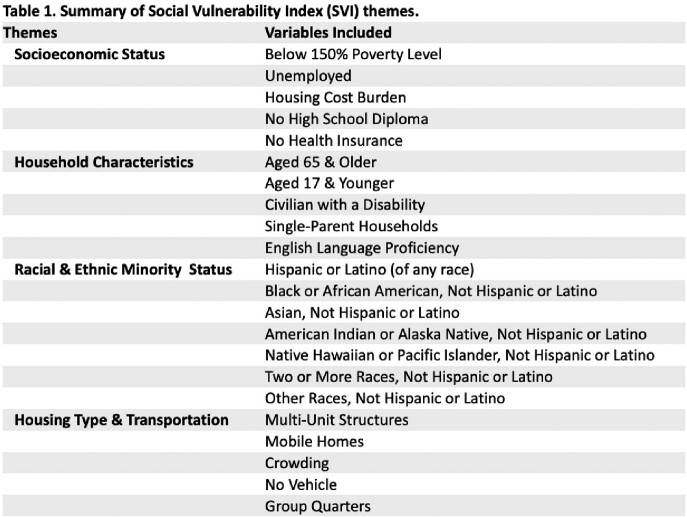

**Results:**

Among the 13,835 residents with CDI, 3,038 (22.0%) had rCDI and 250 (1.8%) had an FMT. Housing type and transportation was the only SVI predictor for rCDI while it was one of several SVI themes predictive of not receiving FMT (Table 2). Adjusting for age and sex, the most vulnerable patients in housing and transportation theme were 13% more likely to have recurrence (P-value<0.01) (Table 3). Independent predictors of FMT receipt included younger age and female sex. Importantly, patients living in a largely minority census tract were 37% less likely to receive an FMT and those in a largely uninsured tract 39% less likely (Table 3). Spatial analysis illustrates a strong discordance between FMT receipt and rCDI rates (Figure 1).

**Figure:**
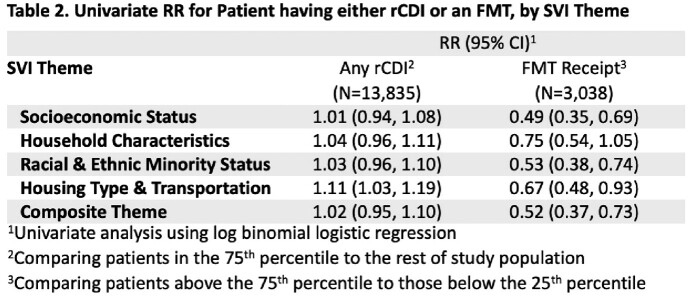

**Figure:**
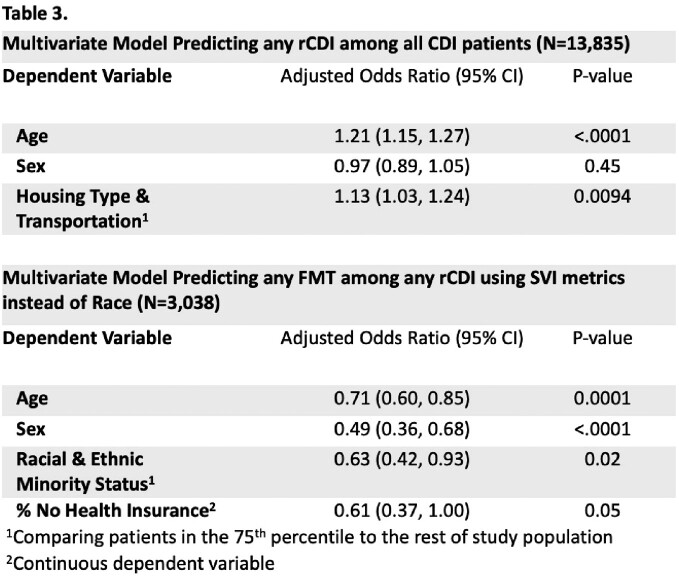

**Figure:**
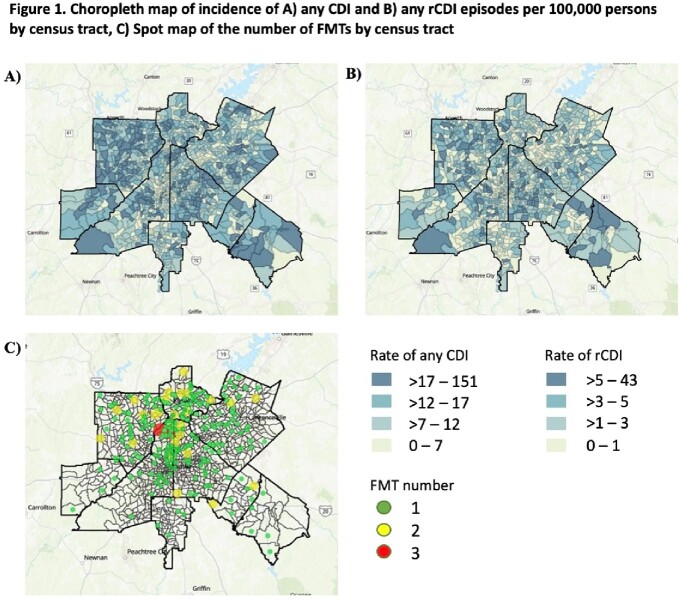

**Conclusion:**

More socially vulnerable populations, defined by access to transportation and the level of household crowding, are more likely to develop rCDI. Likelihood of receiving FMT is substantially reduced among uninsured and persons of black race; both associations need consideration as novel live biotherapeutic products become available.

**Disclosures:**

**Nitin Gupta, MD**, Abbvie: Advisor/Consultant|Amgen: Grant/Research Support|Bristol Myers Squibb: Advisor/Consultant|Bristol Myers Squibb: Grant/Research Support|Janssen: Advisor/Consultant|Janssen: Grant/Research Support|Pfizer/Arena: Advisor/Consultant|Pfizer/Arena: Grant/Research Support

